# Physical and mental health of older people with disabilities in residential homes in Switzerland

**DOI:** 10.1177/20503121211000530

**Published:** 2021-03-10

**Authors:** Monika T Wicki

**Affiliations:** University of Applied Sciences in Special Needs Education Zurich, Zurich, Switzerland

**Keywords:** Gerontology, public health, disabilities, intellectual disability

## Abstract

**Objectives:**

People with disabilities are underserved in terms of health care and prevention, and special health conditions exist among older people with intellectual disabilities. The Swiss Health Survey only covers people over the age of 15 years living in private households. Therefore, this study aims to assess the health status of older persons living in residential facilities for adults with disabilities.

**Methods:**

A cross-sectional survey with written questionnaires was conducted in six cantons in Switzerland to capture context factors and the physical health status of persons aged between 50 and 65 years in residential homes in Switzerland. The survey collected data on physical and mental health from 241 persons aged 50–65 years living in residential homes for people with disabilities. This was compared with data from the 2012 Swiss Health Survey comprising a sample of 2261 persons of the same age with chronic morbidities living in their own apartments.

**Results:**

Regarding their health, 94.1% of the survey respondents rated it as being very good, good or moderate. Although higher limitations on activities of daily living, higher levels of psychological distress and lower energy and vitality were reported by all respondents, a lower level of health issues was assessed than in the sample of persons with chronic morbidities living in their own apartment.

**Conclusion:**

Low energy and vitality, high limitations on activities of daily living, high psychological distress, high obesity rates and the assessment of health issues and pain should be specifically addressed in residential homes for people with disabilities.

## Introduction

People with disabilities are underserved in terms of health care and prevention.^1,2^ They are eight times more likely to report their health as fair or poor than people with no disabilities,^3^ consistently report higher rates of obesity and lack of physical activity, have higher rates of newly diagnosed cases of diabetes and have a three to four times higher rate of cardiovascular disease.^4^ Women with disabilities are less likely to receive timely screening for breast and cervical cancer.^5^ Special health conditions exist among people with intellectual disability (ID). ID, which affects approximately 1%–3% of the population,^6^ covers a wide range of abilities and disabilities, skills and limitations but always includes the following three aspects: (1) a significantly reduced ability to understand new or complex information and to learn and apply new skills, (2) a significantly reduced ability to cope independently, expressed in conceptual, social and practical adaptive skills and (3) early onset (before adulthood), with a lasting effect on development.^7^

In the healthy ageing in ID study, De Winter et al.^8^ found a high prevalence of overweight and obesity in people with ID in the Netherlands, and a higher obesity rate among people with ID than among the general older population (26% vs 10%, respectively). People with ID often have mental illnesses. The risks of depression and of Alzheimer’s disease are well documented.^9,10^ Adults with ID have poorer health than their peers without disabilities.^11^ Challenging behaviour and prescriptions of psychotropic drugs are reported for many persons with ID.^12,13^ There is evidence of a significant level of avoidable suffering due to untreated illnesses.^14,15^ People with ID have a significantly lower mean age at death than those without disabilities.^16,17^ Results of a retrospective study suggest that older adults with Down syndrome (DS) encounter more relocations and are more likely to have their final placement for end-of-life care in a nursing home; in contrast, adults without DS are subjected to less relocation and remain in the same group-home setting.^18^ These findings underline the importance of continuing research on the health status and health service use of people with disabilities and with ID living in residential homes. The data sources available in Switzerland on the life situations of people with disabilities are based on a variety of definitions of disability. For this reason, there is no clear answer to the question of how many people with disabilities there are in Switzerland.^19^

With the enactment of the Swiss Federal Act on the Elimination of Discrimination against People with Disabilities, statistics on the equal rights of people with disabilities were introduced. These equality statistics compare the life situations of people who are classified as having a disability with those of people who are not. The starting point is a medical and social definition of disability: people considered as having a disability are those who state that they have a long-term health problem (medical dimension) and are greatly or somewhat limited in activities of daily living (ADLs; social dimension).

Regarding a person’s well-being, equality statistics provide information on satisfaction with life, health, autonomy, family life, social contacts, residential situation, leisure time and personal safety. Data are drawn for this from different data sets in population statistics. For the health aspect, based on data from the Swiss Statistics on Income and Living Conditions, equality statistics show that most people without disabilities rate their health status as (very) good, whereas slightly less than half of those with disabilities rate their health as good.^3^

For the Swiss Health Observatory (Obsan), Rüesch^20^ examined the health status and health care of people with chronic illnesses who participated in the 2002 Swiss Health Survey and found that people with disabilities used more services in all areas of health care and used these services very intensively.

The Swiss Health Survey only covers the health and health care of persons over the age of 15 years who live in private households. Therefore, the health status and the status of health care in residential facilities are not known. As previous studies have reported a low mean age at death (56 years) in residential homes for people with disabilities in Switzerland, the focus should be on the health status of persons aged 50–65 years.

## Research questions and methods

This research aims at assessing the physical and mental health of people with disabilities aged 50–65 years living in residential homes in Switzerland. The research questions were as follows:

*RQ1*. How do these people rate their general health status?
*RQ2*. How do they perceive their health and activity limitations?
*RQ3*. What intensity of complaints do respondents experience, and what is the prevalence of depression and psychological distress?

### Research design

To capture context factors and the physical health status of people with disabilities in residential homes in Switzerland, we conducted a cross-sectional survey with written questionnaires.

### Sample

The questionnaires were sent to directors of all residential homes for adults with disabilities in four German-speaking cantons (i.e. states – Zurich, Bern, St. Gallen and Luzern), one Italian-speaking canton (Ticino) and one French-speaking canton (Vaud). These six cantons represented 54% of the Swiss population. Swiss residential homes for people with disabilities provided places for 20,000 persons. About 10,000 of the residents were older than 40 years. Residential homes in these six cantons provided places for more than 10,000 persons. Therefore, the sample size necessary for 95% confidence and a margin of error of 5% in these six cantons had to be more than 350 respondents to be representative. Some of these residential homes were specialised for people with specific disabilities (e.g. ID or sensory impairments), while others provided services for people with disabilities in general (i.e. people with different disabilities lived together).

The contact details for the residential homes were provided by the INSOS Switzerland, the Curaviva Switzerland and the website of the Vaud association of residential homes for people with disabilities (Association vaudoise d’institution médico-psycho-sociales). Most of the residential homes for people with disabilities in Switzerland belonged to one of these three associations.

### Sample size

Directors at all 255 residential homes in the six cantons were requested in June 2017 to ask residents aged 50–65 years to voluntarily participate in the study. No exclusion criteria were defined. A letter with explanatory information about the project and the questionnaire were sent, which could also be used to inform legal representatives if needed. Letters and questionnaires were written in German and translated into French and Italian by a professional translator. In July 2017, the directors were contacted by telephone and asked if they had received the letter, if the residents wanted to participate in the survey and whether they needed help interviewing the residents.

In total, directors of 164 residential homes (64% of all residential homes in the six cantons) gave us feedback: in 79 homes, no eligible residents (i.e. aged 50–65 years) wanted to participate, while 31 homes had no eligible residents. Approximately, 910 questionnaires were then sent to the other 54 homes with willing and eligible participants. Respondents voluntarily filled out the questionnaire. Before participating, they filled out an informed consent form, which was written in easily understandable language, where they were adequately informed of the aims, methods, sources of funding, any possible conflicts of interest, institutional affiliations of the researcher, and the anticipated benefits and potential risks of the study. Participants were informed of their right to refuse to participate in the study or to withdraw consent to participate at any time without consequences for themselves. Legal representatives completed the form for any participants who were not able to understand. People with disabilities were only enrolled in the study if they freely agreed to it.

### Indicators

The questions on health status were drawn up in accordance with various items in the Swiss Health Survey, and the use of these questions was permitted by the Federal Office of Public Health. The Swiss Health Survey is based on a comprehensive concept of health. It is strongly oriented to international surveys, such as the European Health Interview, so as to ensure better international comparability.

The first part of our questionnaire contained general questions on age, sex, number of years receiving disability benefits, living arrangements, spouse/partner (yes/no), education/training and nationality. The kind of disability (physical, psychological or intellectual) was self-reported.

All persons answering the questionnaire were persons with chronic morbidity. The item assessing the presence of long-standing health problems (‘Do you have any longstanding illness or health problem?’) had the response options of ‘yes’ or ‘no’. This item is used by the Swiss Health Survey, the European Health Interview Survey (EHIS, HS2 variable) and the European Union Statistics on Income and Living Conditions (EU-SILC, PH020 variable). The EU-SILC survey contains a small module on health, composed of three variables on health status and four variables on unmet health care needs. The variables on health status represent the Minimum European Health Module (MEHM) and measure three different concepts of health: self-perceived health, chronic morbidity (people having a long-standing illness or health problem) and activity limitation (i.e. disability in terms of self-perceived long-standing limitations in usual activities due to health problems). The Swiss Health Survey includes these three concepts, as well as our survey. Therefore, self-rated health was assessed by the question ‘In general, how would you rate your health?’ with the response options ‘very good’ (coded 1), ‘good’ (coded 2), ‘moderate’ (coded 3), ‘bad’ (coded 4) or ‘very bad’ (coded 5). Self-perceived limitations in ADLs were assessed by the MEHM item ‘For at least the past six months, to what extent have you been limited because of a health problem in activities people usually do?’

The response options for the ability to perform ADLs – such as the ability to eat, get up, dress and bathe independently – were on a 4-point scale: ‘yes, without difficulty’ (coded 1), ‘yes, with minor difficulty’ (coded 2), ‘yes, with severe difficulty’ (coded 3) or ‘no’ (coded 4). The Swiss Health Survey and this study recorded general health issues and the intensity of complaints and (physical) pain that respondents experienced – on average – during the 4 weeks prior to the interview: back pain, fatigue, stomach ache, diarrhoea or constipation, sleep disturbance, headache, heart rhythm disturbance, chest pain and fever or pain in the shoulders or arms. The response options were ‘not at all’, ‘somewhat’ or ‘strong’.

Prevalence of depression was assessed with the Patient Health Questionnaire (PHQ-9). PHQ-9 is the validated major depressive disorder module of the full PHQ and is used to provisionally diagnose depression and grade of severity of symptoms in general medical and mental health settings. On a 4-point Likert-type scale (not at all = 0; several days = 1; more than half the days = 2; nearly every day = 3), respondents reported if they had been bothered by specific problems over the last 2 weeks. A score of 1–4 indicated minimal depression, 5–9 mild depression, 10–14 moderate depression, 15–19 moderately severe depression and 20–27 severe depression.

In the Swiss Health Survey and in our survey, the Mental Health Inventory (MHI) was used. The MHI is a method for evaluating mental health issues such as anxiety, depression, behavioural control, positive affect and general distress, and it helps in the measure of overall emotional functioning.^21^ Respondents to our questionnaire answered nine questions: ‘During the past four weeks, how much of the time . . .’ (1) ‘Has your daily life been full of things that were interesting to you?’, (2) ‘Have you been a very nervous person?’, (3) ‘Have you felt so down in the dumps that nothing could cheer you up?’, (4) ‘Have you felt calm and peaceful?’, (5) ‘Have you felt cheerful, light-hearted?’, (6) ‘Have you felt downhearted and blue?’, (7) ‘Have you been moody, or brooded about things?’, (8) ‘Were you a happy person?’ or (9) ‘Have you been in low or very low spirits?’ Respondents answered on a 5-point Likert-type scale, with 1–5 being ‘all of the time’, ‘most of the time’, ‘some of the time’, ‘a little of the time’ and ‘none of the time’, respectively.

From these nine questions, we calculated the MHI-5 score and the Energy and Vitality Index (EVI).^22^ This scale is a part of the EHIS and captures the positive side of mental health. Body mass index (BMI, a measure of body fat for adult men and women) was assessed based on height and weight of the person.

The questionnaire used in this study was not pilot tested. As most questions used were several times tested within other questionnaires (Swiss Health Survey and EHIS), we could assume that the questions had been adequately examined.

People with disabilities who wanted to participate filled out the questionnaire or were interviewed by carers or students. At the end of the questionnaire, respondents were asked if they had filled out the questionnaire on their own or if they had received help. Questions on health service use and social networks were also asked in the questionnaire, but these questions are not addressed in this article.

### Analysis

We analysed self-rated health as a dichotomous measure: ‘very good’, ‘good’ or ‘moderate’ were coded as 0 and ‘bad’ or ‘very bad’ as 1. ADLs and instrumental activities of daily living (IADLs; e.g. cooking or cleaning windows) were handled in accordance with the 2012 Swiss Health Survey by forming four groups: a person answering 1 for all activities was assigned to group 1, ‘no difficulty performing these activities’; a person answering 2 for at least one activity was assigned to group 2, ‘minor difficulty performing at least one of these activities’; a person answering 3 for at least one activity was assigned to group 3, ‘severe difficulty performing at least one of these activities’ and a person answering 4 for at least one activity was assigned to group 4, ‘cannot perform at least one of these activities’.

As in the Swiss Health Survey (2012), the responses of questionnaire respondents who answered all of the questions on the different kinds of complaints and who had no fever were added together. Three categories were formed based on the sum total: fewer than 10 points (no or hardly any pain and symptoms), 10–12 points (some pain and symptoms) and more than 12 points (strong pain and symptoms).

For the EVI score, we used questions 1, 5, 7 and 9 of the MHI. Questions 1 and 5 were recoded 1 = 6, 2 = 5, 3 = 3.5, 4 = 2 and 5 = 1, and questions 7 and 9 were recoded 1 = 1, 2 = 2, 3 = 3.5, 4 = 5 and 5 = 6. The points for the four questions were added up and then divided into 20 and multiplied by 100 (following the guidelines for the indices of the Swiss Health Survey). These four items measured a person’s energy level and fatigue; higher scores indicated higher vitality. EVI scores were categorised as follows: high energy and vitality (more than 70), moderate energy and vitality (between 63 and 70) and low energy and vitality (lover than 63).

The MHI-5 Items is a brief, valid and reliable international instrument for assessing mental health in adults.^23^ The items are from the EHIS and are used in the Swiss Health Survey and in our survey. Response options are on a 5-point Likert-type scale ranging from ‘always’ to ‘never’. We recoded these as was done for the Swiss Health Survey. The score for each individual therefore ranged from 5 to 30. This was then transformed into a variable ranging from 0 to 100 using a standard linear transformation. A score of 100 represented optimal mental health. Lower scores on these five MHI items indicated higher psychological distress. Although this indicated a greater probability that a person might have an anxiety disorder or a depressive disorder, it was not a diagnosis. MHI-5 scores were categorised as follows: high psychological distress (lower than 52), moderate distress (between 52 and 72) and low distress (higher than 72).

We compared the state of health of persons with a lifelong disability (disability benefits since childhood) and persons affected by illness or accident (disability benefits after the age of 20 years). We also compared the group of people with ID and the group of people with other disabilities (physical, sensory or psychological). Furthermore, the responses of people living in residential homes were compared with people without disabilities living in their own apartments. For comparison with the general population, we used data from the 2012 Swiss Health Survey. These data were made available to us by the Federal Office of Public Health.

Data analyses were performed using the IBM SPSS Statistics 25.0. Chi-square tests and the Mann–Whitney *U* test were used for nominal and ordinal variables and *t*-tests for metric variables to assess whether differences between groups were statistically significant. *p*-values of less than 0.05 were regarded as statistically significant. The different questions were validated with principal component and factor analysis.

An approval from the responsible cantonal ethics committee was sought (BASEC-Nr. Req-2020-00887). The answer was: the research project does not fall within the scope of the Human Research Act (HRA). Therefore, an authorisation from the ethics committee was not required. The study followed the Recommendations for the Protection of Research Participants of the International Committee of Medical Journal Editors and the Declaration of Helsinki – Ethical Principles for Medical Research Involving Human Subjects of the World Medical Association. Informed consent was sought by participants as well as by their legal representatives. The detailed responses were anonymised by the residential homes (responses were numbered, and participants’ names were removed) and by the researcher (birth date was replaced with age).

## Results

### Characteristics of the population

241 residents (123 men and 118 women) aged 50–65 years were surveyed in 54 residential homes. Most of the surveyed residents (88.8%) lived in homes in the German-speaking part of Switzerland; 94.9% were born in Switzerland; 84.7% had not completed basic vocational training; 17.8% had a live-in partner.

Of the 145 residents with ID, 115 residents had ID only and 30 residents had ID and another disability. The 96 residents without ID had other (multiple) disabilities. 79 persons had physical/sensory disabilities, and 77 persons had psychological disabilities.

The mean age of the survey respondents was 56.9 years (standard deviation (SD) = 4.39 years). The mean number of years that respondents had been receiving disability benefits was 36.2 (SD = 16.35 years); 61.8% of the residents had received disability benefits since childhood. For 79 respondents, it was not known how long they had been receiving disability benefits (Table 1).

**Table 1.** Characteristics of persons living in residential homes.

	*n*	%
Region		
German speaking	214	88.8
Romandie/Tessin	27	11.2
Age		
M	56.93 years	
SD	4.39 years	
Sex		
Man	123	51.0
Woman	118	49.0
Vocational training		
<2 years basic training	182	84.7
>2 years basic training (missing: 26)	33	15.3
Disabilities (multiple responses)		
Physical/sensory	79	32.8
Psychological	77	32.0
Intellectual	145	60.2
Supported answering the questionnaire		
Yes	196	81.3
Disability pension since		
M (missing: 79)	36.22 years	
SD	16.35 years	

M: mean; SD: standard deviation.

From the Swiss Health Survey, data were extracted on 2261 persons aged 50–65 years who had answered yes to the question on chronic illness.

### Health status

Almost all respondents living in residential homes (94.1%) rated their general health status as very good, good or moderate, while only a small number (5.9%) rated their general health status as bad or very bad. More respondents with lifelong disability rated their health as very good, good or moderate (96.3%) than those who started receiving their disability benefits during adulthood (86%; Fisher–Yates *t*-test, *p* = 0.036). People with ID and people with other disabilities rated their health status equally often as very good, good or moderate.

People with disabilities living in residential homes for adults with disabilities (our survey respondents) rated their general health status better than people with chronic morbidities living in their own apartments (2012 Swiss Health Survey respondents): only 86% of the people with chronic morbidities living in their own apartments rated their general health status as very good, good or moderate (Fisher–Yates *t*-test, *p* = 0.043; see Table 2).

**Table 2.** Self-rated health.

	Very good, good or moderate		Bad or very bad		*p*
	*n*	%	*n*	%	
Residential home (*n* = 237)	223	94.1	14	5.9	
Own apartment, with chronic morbidity (*n* = 2261)*	1945	86	316	14	0.043
Lifelong disability (*n* = 81)	78	96.3	3	3.7	
Disability as adult (*n* = 50)	43	86.0	7	14.0	0.036
Persons with intellectual disabilities (*n* = 143)	134	93.7	9	6.3	
Persons with other disabilities (*n* = 94)	89	94.7	5	5.3	0.5

* Swiss Health Survey 2012.

### Self-perceived limitations

Forty-seven persons in residential homes (21.2%) said that they were severely limited; 88 (39.6%) persons said that they were limited but not severely, while 19 persons refused to answer the question. No differences in self-perceived limitations were found between those with lifelong disability and those who started receiving their disability benefits during adulthood, between those with ID and those with other disabilities or between those living in residential homes and those with chronic morbidity and living in their own apartments (Table 3).

**Table 3.** Self-perceived limitations.

	Severely restricted		Somewhat restricted		Not restricted		*p*
	*n*	%	*n*	%	*n*	%	
Residential home (*n* = 222)	47	21.2	88	39.6	87	39.2	
Own apartment, with chronic morbidity* (*n* = 2264)	347	15.3	914	40.4	1003	44.3	0.061
Lifelong disability (*n* = 77)	20	26	27	35.1	30	39	
Disability as adult (*n* = 48)	11	22.9	22	45.8	15	31.3	0.478
Persons with intellectual disabilities (*n* = 137)	33	24.1	47	34.3	57	41.6	
Persons with other disabilities (*n* = 85)	14	16.5	41	48.2	30	35.3	0.103

* Swiss Health Survey 2012.

### Difficulties performing ADLs

A total of 216 respondents living in residential homes for people with disabilities were able to eat independently without difficulty or with minor difficulty (177 persons, 73.8% and 39 persons, 16.3%, respectively); 10% of the respondents could only eat independently with severe difficulty or not at all (each category: 12 persons, 5%).

Regarding IADLs, 97 respondents (40.4%) were able to cook their meals independently with no or minor difficulty, and 142 persons had severe difficulty or were not able to cook (42 persons, 17.5% and 100 persons, 41.7%, respectively). 161 respondents (67.4%) were not able to manage their finances (Table 4).

**Table 4.** Activities of daily living (*N* = 241).

	Without difficulty	With minor difficulties	With severe difficulties	Not able to manage independently	*p*
Activities of daily living					
Eat (people with other disabilities, *n* = 95)	80 (84.2%)	9 (9.5%)	1 (1%)	5 (5.3%)	0.008
Eat (people with ID, *n* = 145)	97 (66.9%)	30 (20.7%)	11 (7.6%)	7 (5%)	
Get up (people with other disabilities, *n* = 95)	80 (84.2%)	10 (10.5%)	0 (0%)	5 (5%)	0.008
Get up (people with ID, *n* = 145)	97 (66.8%)	21 (14.5%)	8 (0.6%)	19 (13.1%)	
Dress (people with other disabilities, *n* = 95)	71 (74.7%)	19 (20%)	0 (0%)	5 (5%)	0.001
Dress (people with ID, *n* = 145)	83 (57.2%)	25 (17.2%)	12 (8.2%)	25 (17.2%)	
Go to the toilet (people with other disabilities, *n* = 95)	86 (90.5%)	4 (4.2%)	0 (0%)	5 (5%)	0.000
Go to the toilet (people with ID, *n* = 145)	86 (59.3%)	20 (13.8%)	8 (0.6%)	31 (21.4%)	
Bath (people with other disabilities, *n* = 95)	62 (65.3%)	23 (24.2%)	3 (3.2%)	7 (7.4%)	0.000
Bath (people with ID, *n* = 145)	57 (39.3%)	32 (22.6%)	12 (8.3%)	44 (30.3%)	
Instrumental activities of daily living					
Cook (people with other disabilities, *n* = 95)	32 (33.6%)	24 (25.2%)	8 (8.4%)	31 (32.6%)	0.000
Cook (people with ID, *n* = 145)	11 (7.6%)	30 (20.7%)	34 (23.4%)	69 (47.6%)	
Phone (people with other disabilities, *n* = 94)	58 (61.1%)	15 (15.8%)	5 (5%)	16 (16.8%)	0.000
Phone (people with ID, *n* = 145)	43 (29.7%)	24 (16.6%)	21 (14.4%)	57 (39.3%)	
Shopping (people with other disabilities, *n* = 95)	52 (54.7%)	16 (16.8%)	10 (10.5%)	17 (817.9%)	0.000
Shopping (people with ID, *n* = 144)	32 (22.2%)	25 (17.4%)	26 (18.1%)	61 (42.4%)	
Make the laundry (people with other disabilities, *n* = 92)	35 (38.1%)	14 (15.2%)	5 (5.4%)	38 (41.3%)	0.000
Make the laundry (people with ID, *n* = 140)	15 (10.7%)	11 (7.9%)	13 (9.3%)	101 (72.1%)	
Doing easy housework (people with other disabilities, *n* = 94)	49 (52.1%)	21 (22.3%)	12 (12.8%)	12 (12.8%)	0.000
Doing easy housework (people with ID, *n* = 144)	43 (29.9%)	30 (20.8%)	28 (19.4%)	43 (29.9%)	
Doing strong housework (people with other disabilities, *n* = 88)	23 (26.1%)	15 (17.4%)	14 (15.9%)	36 (40.9%)	0.000
Doing strong housework (people with ID, *n* = 141)	6 (4.1%)	19 (13.2%)	27 (18.8%)	89 (61.8%)	
Managing finances (people with other disabilities, *n* = 95)	20 (21.1%)	17 (17.9%)	9 (9.5%)	49 (51.6%)	0.000
Managing finances (people with ID, *n* = 144)	1 (0.6%)	9 (6.3%)	22 (15.3%)	112 (77.8%)	
Using public transportation (people with other disabilities, *n* = 95)	45 (47.4%)	16 (16.8%)	7 (7.4%)	27 (18.4%)	0.000
Using public transportation (people with ID, *n* = 145)	29 (20%)	25 (17.2%)	20 (13.8%)	71 (48.9%)	

ID: intellectual disability.

Of all the respondents living in residential homes for people with disabilities, 46.1% had no difficulty performing any ADLs and 21.7% were not able to manage at least one ADL. Of those with ID living in residential homes, 31% were not able to manage at least one ADL, while only 7.4% of persons with other disabilities were not able to manage at least one ADL. This difference was significant (*χ*^2^ [1, *N* = 240] = 11.959, *p* = 0.001).

People aged 50–65 years with chronic illnesses living in their own apartments who participated in the 2012 Swiss Health Survey were less limited than people of the same age living in residential homes for people with disabilities: 85.8% of them reported having no difficulties performing any ADLs (*χ*^2^ [1, *N* = 1422] = 201.331, *p* = 0.000). The same differences could be found for IADLs.

### General health issues and pain

The most frequently mentioned complaints were fatigue, diarrhoea or constipation, sleep disturbances and headache: 11.7% of the respondents had experienced strong fatigue in the previous 4 weeks, 10.6% had experienced strong diarrhoea or constipation, 7.5% had experienced strong sleep disturbances and 4.9% had experienced strong headaches.

Of all those living in residential homes for people with disabilities, 27.9% reported no or hardly any complaints or pain, while 38.5% reported strong complaints and pain. People with ID reported strong complaints and pain significantly less often (32%) than those with other disabilities (48.2%; median in both groups = 2, Mann–Whitney *U* test: *z* = −2.020, *p* ⩽ 0.05). No difference was found between those living in residential homes and those with chronic morbidities living in their own apartments (Table 5).

**Table 5.** General health issues and pain.

	No or hardly any complaints and pain	Some complaints and pain	Strong complaints and pain	*p*
Residential homes (*n* = 208)	58 (27.9%)	70 (33.7%)	80 (38.5%)	
Own apartment, with chronic morbidity (*n* = 2115)*	658 (31.1%)	658 (31.1%)	799 (37.8%)	0.062
Persons with intellectual disabilities (*n* = 125)	38 (30.4%)	47 (37.6%)	40 (32%)	
Persons with other disabilities (*n* = 83)	20 (24.1%)	23 (27.7%)	40 (48.2%)	0.043

* Swiss Health survey 2012.

### Aspects of mental health

When asked if they had experienced a recent decline in their ability to memorise new things, 30.2% of the respondents answered yes.

215 persons living in residential homes for people with disabilities answered the MHI questions. Regarding energy and vitality (EVI), 47.9% reported having low vitality. People with chronic illnesses living in their own apartments (Swiss Health Survey 2012 respondents) reported having significantly higher vitality: only 33.5% reported having low vitality (Mann–Whitney *U* test: *z* = −4.913, *p* ⩽ 0.001). People with ID and people with other disabilities living in residential homes had no differences in vitality (Table 6).

**Table 6.** Mental health.

	MHI: EVI	Mann–Whitney *U*		MHI-5	Mann–Whitney *U*		PHQ-9	Mann–Whitney *U*	
		*Z*	*p*		*Z*	*p*		*Z*	*p*
	Low vitality (0–62 points)			High psychological distress (<52 points)			Moderate to severe depression (10–27 points)		
Residential homes (EVI: *n* = 215; MHI-5: *n* = 213; PHQ-9: *n* = 200)	103 (47.9%)			11 (5.2%)			22 (11%)		
Own apartment, with chronic morbidity (EVI: *n* = 2161; MHI-5: *n* = 2147)*	724 (33.5%)	−4.913	0.001	55 (2.6%)	−4.373	0.000	NA	NA	NA
Lifelong disability (EVI: *n* = 71; MHI-5: *n* = 70; PHQ-9: *n* = 66)	27 (38%)			5 (7.1%)			5 (7.6%)		
Disability as adult (EVI: *n* = 48; MHI-5: *n* = 49; PHQ-9: *n* = 46)	30 (62.5%)	−1.965	0.049	8 (16.3%)	−2.977	0.003	7 (15.2%)	−1.281	0.2
Persons with intellectual disabilities (EVI: *n* = 129; MHI-5: *n* = 127; PHQ-9: *n* = 120)	58 (45%)			14 (11%)			12 (10%)		
Persons with other disabilities (EVI: *n* = 86; MHI-5: *n* = 86; PHQ-9: *n* = 80)	45 (52.3%)	−0.584	0.56	13 (15.1%)	−1.629	0.103	10 (12.5%)	−0.552	0.581

EVI: Energy and Vitality Index; MHI: Mental Health Inventory; PHQ: Patient Health Questionnaire; NA: not available.

* Swiss Health Survey 2012.

5.2% of the respondents in residential homes reported high psychological distress. People living in residential homes for people with disabilities had a higher level of psychological distress than those with chronic illnesses living in their own apartments: 91.6% of persons living in residential homes reported high (5.2%) or moderate (86.4%) psychological distress whereas only 80.9% of those with chronic illnesses living in their own apartments reported high (2.6%) or moderate (77.9%) psychological distress (Mann–Whitney *U* test: *z* = −4.373, *p* ⩽ 0.001).

According to the PHQ-9 assessment, 11% of those living in residential homes for people with disabilities had moderate or severe depression. The 2012 Swiss Health Survey did not use the PHQ-9 instrument. Therefore, it was not possible to compare our data with the data from the Swiss Health Survey. But 11% represents a high prevalence, compared with the results of a German study^24^ that found moderately severe to very severe levels of depression in 5.6% of the general population (age 15 to over 90 years). No difference was found in prevalence of depression between persons with ID and persons with other disabilities.

### BMI

Overweight and obesity were highly prevalent (55.7%) among all persons living in residential homes for people with disabilities. Within the general Swiss population in 2017, 42% were overweight or obese.^25^ Participants in the 2012 Swiss Health Survey with chronic illnesses, living in their own apartments and aged 50–65 years were as frequently overweight (57.1%, 1286 persons) as people of the same age living in residential homes for people with disabilities (Table 7).

**Table 7.** Overweight and obesity (BMI).

(Missing: 56)			
	Underweight (<18)	Healthy weight (18–25)	Overweight (>24.9)
Residential home (*n* = 185)	9 (4.9%)	73 (39.5%)	103 (55.7%)
Own apartment, with chronic morbidity (*n* = 2251)*	46 (2%)	919 (40.8%)	1286 (57.1%)
Lifelong disability (*n* = 81)	4 (6.3%)	29 (45.3%)	31 (48.4%)
Disability as adult (*n* = 50)	2 (4.4%)	17 (37.8%)	26 (57.8%)
Persons with intellectual disabilities (*n* = 113)	4 (3.5%)	45 (39.8%)	64 (56.6%)
Persons with other disabilities (*n* = 72)	5 (6.9%)	28 (38.9%)	39 (54.2%)

* Swiss Health Survey 2012.

No differences in BMI were found between people with ID and those with other disabilities, or between those with lifelong disabilities and those who started receiving disability benefits in adulthood.

## Discussion

The main findings on the health status of people with disabilities living in special residential homes are threefold: (1) almost all the persons living in residential homes for people with disabilities rated their health as being very good, good or moderate; (2) people with disabilities living in residential homes were highly limited in ADLs and IADLs and those with ID were the most limited; and (3) people living in residential homes for people with disabilities experienced low vitality but high psychological distress: 47.9% reported low vitality, 5.2% reported high psychological distress and a high prevalence of depression was found.

Regarding finding (1), in a study conducted in Scotland on the general health status of people with and without IDs and the extent of health-related limitations on daily activities, Hughes-McCormack et al.^26^ found that a greater proportion of adults with IDs (40.3%) reported poor health than the general population (13.8%). In our survey, 5.9% of those aged 50–65 years living in residential homes rated their health as poor or very poor. In the general population in the same age group, 14% of those with chronic morbidities (316 persons) rated their health as poor or very poor.

This study shows that people with chronic morbidities living in their own apartments rated their health as poor or very poor significantly more often than persons with chronic morbidities living in a residential home for persons with disabilities; however, regarding finding (2), people living in residential homes experienced significantly more limitations on ADLs than people with chronic morbidities living in their own apartment. The most limited were persons with ID. Of those persons with chronic morbidities living in their own apartment, only 1.1% were not able to manage at least one ADL, and 15.6% were not able to manage at least one IADL.

King et al.^27^ also found a greater ability to perform ADLs and IADLs among those living in independent or community group-home settings compared with those living in traditional residential settings. The epidemiological study of Oppewal et al.^28^ reported a clear decline in the daily functioning of older adults with ID over a 3-year follow-up period.

People living in residential homes (our survey respondents) and those in the same age group with chronic morbidity living in their own apartments (Swiss Health Survey 2012) reported the same extent of self-perceived limitations, although their limitations in daily life were significantly different. Care providers in residential homes should be aware of this and focus on maintaining residents’ independence as much as possible, while care providers for persons with chronic morbidities living in their own apartments should be aware that unmet needs for assistance with ADLs are associated with increased hospitalisation and mortality risks.^29^

People with ID reported strong complaints and pain significantly less often than persons with other disabilities. Previous studies on the ID population have reported that symptoms and pain are often not recognised or managed well, pain relief is inadequate and chronic pain is highly prevalent due to associated physical disabilities.^21,30^

Connolly et al.^31^ showed that the factors with the highest impact on ADL disability after age 65 years were pain, BMI and taking five or more medications. De Winter et al.^8^ found overweight and obesity to be highly prevalent in people with ID, with more obesity (26%) than in the general Dutch older population (10%). Also, this study showed that overweight and obesity were highly prevalent (55.7%) among persons aged 50–65 years living in residential homes for people with disabilities, while only 42% of the general Swiss population are overweight or obese (Swiss Health Survey 2017).^25^

Furthermore, regarding finding (3), people living in residential homes for people with disabilities experienced low energy and vitality but high psychological distress and a high prevalence of depression. People with chronic morbidities of the same age group living in their own apartment reported significantly higher vitality and lower psychological distress. This result is contrary to the negative influences on mental health identified in other literature: unemployment, low education level, a short duration of disability, low self-rated health and low levels of social support.^32^ Perceived disability-related discrimination is associated with depression, while poor self-rated health and psychological distress are linked with poorer well-being.^33^ The low vitality and high psychological distress levels of those living in residential homes are alarming, and more research on this issue has to be conducted.

People with ID often have mental illnesses. The risk of depression is well documented.^9,10^ In cases where mental comorbidities such as depression or anxiety disorders develop, personal resilience is an important factor. Systematic reviews have reported negative associations between resilience and symptoms of mental distress in patients with chronic diseases.^34^ It is important to strengthen personal resilience and psychological competence for older people with disabilities living in residential homes.

### Study limitations

For a representative study with 95% confidence and a margin of error of 5% in the six chosen cantons, more than 350 respondents were necessary. Only 241 persons responded to the questionnaires. Thus, the results cannot be generalised for all persons aged 50–65 years living in residential homes in Switzerland. The large differences within regions in Switzerland (e.g. in population densities, rural or urban situations and health and disability care services) should also be considered.

This study only provides data on the health status of people with disabilities living in four German-speaking cantons in Switzerland. Because only a few questionnaires were completed in two of the other cantons (Ticino and Vaud), the results for these two cantons cannot be generalised.

It is important to discuss the fact that almost all the participants rated their health as being good, in spite of clear evidence to the contrary. Also, Likert-type scales were used to assess several aspects in this survey. Hartley and MacLean^35^ found that Likert-type scales should include pictorial representations of response alternatives, a single set of one or two word response descriptors and clarifying questions. In this study, the questions were written in easy-to-read language; response alternatives with pictures were not provided.

Special challenges associated with obtaining self-reports from people with ID include proxy responding on behalf of the person with ID, which involves the provision of responses by relatives or someone who knows the person well. Concerns about the validity of such data are mentioned, as to whether they accurately reflect the feelings of the individual.^36^

## Conclusion

The results of this study indicate possible important differences in the health status of people living in residential homes compared with those with chronic illnesses living in their own apartments and compared with the general population in Switzerland. This study also provides information on possible differences between the health status of older people with ID living in residential homes and those with other disabilities.

These findings have implications for health service provision and for staff providing care to people with disabilities living in residential homes. Pain assessment in residential homes for people with ID is important. Low energy and vitality, high limitations on ADLs, high psychological distress levels and high obesity rates should be addressed specifically. Physical fitness programmes could be implemented for older adults with ID, and it is important to raise people’s awareness of their rights and of the social possibilities and services available to them, to provide them with mental health help and to engage in extensive social activities aimed at providing resources to persons with disabilities. Personal resilience and psychological competence to deal with and go beyond traumatic or stressful events should be strengthened. Further analysis will be conducted on the question of health service provision and health service use of persons with disabilities living in residential homes.

## Supplemental Material

sj-pdf-1-smo-10.1177_20503121211000530 – Supplemental material for Physical and mental health of older people with disabilities in residential homes in Switzerland Click here for additional data file. Supplemental material, sj-pdf-1-smo-10.1177_20503121211000530 for Physical and mental health of older people with disabilities in residential homes in Switzerland by Monika T Wicki in SAGE Open Medicine

sj-pdf-2-smo-10.1177_20503121211000530 – Supplemental material for Physical and mental health of older people with disabilities in residential homes in Switzerland Click here for additional data file. Supplemental material, sj-pdf-2-smo-10.1177_20503121211000530 for Physical and mental health of older people with disabilities in residential homes in Switzerland by Monika T Wicki in SAGE Open Medicine
