# Supplementary material for: Physical and mental health of older people with disabilities in residential homes in Switzerland
Source: SAGE Open Med. 2021 Mar 10;9:20503121211000530. doi: 10.1177/20503121211000530 (PMC7958166; doi:10.1177/20503121211000530)
Supplement: sj-pdf-1-smo-10.1177_20503121211000530 – Supplemental material for Physical and mental health of older people with disabilities in residential homes in Switzerland [file sj-pdf-1-smo-10.1177_20503121211000530.pdf]

Interkantonale Hochschule
für Heilpädagogik

Die Gesundheit von Menschen mit Beeinträchtigung in der Schweiz

Verschiedene Behörden und Institutionen für Menschen mit Beeinträchtigung möchten wissen:

- Gehen Menschen mit Beeinträchtigung in der Schweiz zum Arzt, wenn sie krank sind?
- Bekommen sie die Behandlung, die sie brauchen?
- Gibt es dabei Unterschiede zu Menschen ohne Beeinträchtigung?
- Brauchen Menschen mit Beeinträchtigung mehr Unterstützung, wenn sie zum Arzt gehen?

Sie erhalten deshalb von uns diesen Fragebogen. Im Fragebogen sind 46 Fragen:

- Fragen zu Ihrer Person
- Fragen zu Ihrer Wohnsituation
- Fragen zu Ihrer IV-Rente und wie viel Unterstützung Sie brauchen
- Fragen zu Ihrer Gesundheit
- Fragen zu Arztbesuchen und Spitalaufenthalten

Bitte füllen Sie den Fragebogen **vollständig** aus. Sie können auch jemanden aus Ihrem Umfeld bitten, Sie zu unterstützen.

Wenn Sie Fragen haben, rufen Sie uns bitte an. Frau Monika Wicki hilft Ihnen gerne weiter.
Telefon: 044 317 12 34

Datenschutz

Für diese Studie gilt der **Datenschutz**. Das heisst: Ihre Antworten werden nur für diese Studie verwendet. Wir geben Ihre Antworten **nicht** an andere Personen weiter. Wir dürfen auch **nicht** mit anderen über Ihre Antworten sprechen. Ihre Antworten sind **anonym**, das heisst: Niemand weiss Ihren Namen. Und Ihre Daten werden **verschlüsselt**. Das heisst: Nur wir können Ihre Daten lesen.

Fragen zu Ihrer Person

1 An welchem Datum wurden Sie geboren?

____

2 Geschlecht

◯ Mann

◯ Frau

3 Wo wurden Sie geboren?

◯ In der Schweiz

◯ Im Ausland

◯ Weiss nicht

4 Was ist Ihre Muttersprache?

◯ Deutsch

◯ Französisch

◯ Italienisch

◯ Rätoromanisch

◯ Eine andere Sprache

Welche Sprache?__________________________________

◯ Weiss nicht

5 Welches ist die höchste Ausbildung, die Sie abgeschlossen haben?

Die Antworten gelten für die Schweiz.

Haben Sie Ihre Ausbildung im Ausland gemacht? Dann wählen Sie die Antwort, die am ehesten zu Ihnen passt.

- ◯ Ich habe die Pflicht-Schulzeit **nicht** abgeschlossen.
- ◯ Ich habe die Pflicht-Schulzeit abgeschlossen,
  aber ich habe keine weitere Ausbildung gemacht.
- ◯ Ich habe eine **Grundbildung** in einem Beruf gemacht.
  Ich habe ein Eidgenössisches Berufs-Attest EBA.
- ◯ Ich habe eine **Lehre** in einem Beruf gemacht.
  Ich habe ein Eidgenössisches Fähigkeits-Zeugnis EFZ.
- ◯ Ich habe eine Ausbildung gemacht, die von der
  Invaliden-Versicherung (IV) bezahlt wurde.
  Zum Beispiel die Praktische Ausbildung nach Insos.
- ◯ Ich habe das **Gymnasium** abgeschlossen und die Matur gemacht.
- ◯ Ich habe eine Fach-Mittelschule abgeschlossen.
- ◯ Ich habe ein eidgenössisches Diplom oder einen eidgenössischen Fach-Ausweis.
- ◯ Ich habe eine höhere Fachschule abgeschlossen.
- ◯ Ich habe eine Hochschule abgeschlossen.
- ◯ Weiss nicht.

Schaffhauserstrasse 239
Postfach 5850
CH-8050 Zürich
T +41 44 317 12 34
F +41 44 317 11 10
wicki.monika@hfh.ch
www.hfh.ch

Fragen zu Ihrer Wohnsituation

6 Wie wohnen Sie?

- ◯ In einer eigenen Wohnung
- ◯ Bei Angehörigen
- ◯ In einer Einrichtung für Menschen mit Beeinträchtigung
- ◯ In einem Pflegeheim
- ◯ Anders, zum Beispiel in einer betreuten Wohngemeinschaft

Wie genau? ____

7 Haben Sie einen Lebenspartner oder eine Lebenspartnerin?

- ◯ Ja
- ◯ Nein

8 Wer unterstützt Sie im Alltag?

Sie können mehrere Antworten ankreuzen.

- ◯ Jemand von der IV
- ◯ Angehörige oder Partner/Partnerin
- ◯ Pflegeperson, Betreuung oder Spitex
- ◯ Andere Personen, zum Beispiel ein Nachbar oder ein Freund

Wer? ____

- ◯ Niemand

**9 Wenn Sie im Alltag unterstützt werden:
Wie zufrieden sind Sie mit der Unterstützung?**

- ◯ Sehr zufrieden
- ◯ Eher zufrieden
- ◯ Eher nicht zufrieden
- ◯ Nicht zufrieden
- ◯ Weiss nicht

10 Wie zufrieden sind Sie mit Ihrer jetzigen Wohnsituation?

- ◯ Sehr zufrieden
- ◯ Eher zufrieden
- ◯ Eher nicht zufrieden
- ◯ Nicht zufrieden
- ◯ Weiss nicht

11 Können Sie eine Person in Ihrem Umfeld um Unterstützung bitten, zum Beispiel jemanden in Ihrer Familie, Ihren Partner oder Ihre Partnerin oder einen Nachbarn oder eine Nachbarin?

- ◯ Ja, mehrere Personen
- ◯ Ja, eine Person
- ◯ Nein
- ◯ Weiss nicht

Fragen zu Ihrer IV-Rente und wie viel Unterstützung Sie brauchen

12 Weshalb haben Sie eine IV-Rente?

Sie können mehrere Antworten ankreuzen.

- ○ Ich habe eine körperliche Beeinträchtigung.
- ○ Ich habe eine psychische Beeinträchtigung.
- ○ Ich habe eine kognitive oder geistige Beeinträchtigung.
- ○ Anderer Grund:____
- ○ Weiss nicht

13 Wie lange bekommen Sie die IV-Rente schon?

Seit dem Jahr ______________.

14 Was können Sie selbstständig und ohne Unterstützung?

Kreuzen Sie bei jeder Tätigkeit eine Antwort an.

	Ohne Mühe	Mit etwas Mühe	Mit viel Mühe	Geht nicht
Essen	○	○	○	○
Ins Bett steigen, aus dem Bett steigen, von einem Sessel aufstehen	○	○	○	○
Sich anziehen und ausziehen	○	○	○	○
Zur Toilette gehen	○	○	○	○
Baden oder duschen	○	○	○	○

15 Was können Sie selbstständig und ohne Unterstützung?

Kreuzen Sie bei jeder Tätigkeit eine Antwort an.

	Ohne Mühe	Mit etwas Mühe	Mit viel Mühe	Geht nicht
Essen zubereiten	◯	◯	◯	◯
Telefonieren	◯	◯	◯	◯
Einkaufen	◯	◯	◯	◯
Wäsche waschen	◯	◯	◯	◯
Leichte Hausarbeit erledigen	◯	◯	◯	◯
Gelegentlich schwere Hausarbeit erledigen	◯	◯	◯	◯
Sich um Finanzen kümmern	◯	◯	◯	◯
Die öffentlichen Verkehrsmittel benützen	◯	◯	◯	◯

Fragen zu Ihrer Gesundheit

16 Welche dieser Beschwerden hatten Sie in den letzten vier Wochen?

Kreuzen Sie bei allen Beschwerden eine Antwort an.

	Überhaupt nicht	Ein bisschen	Stark
Rückenschmerzen oder Kreuzschmerzen	○	○	○
Müdigkeit, Schwächegefühl	○	○	○
Schmerzen oder Druckgefühl im Bauch	○	○	○
Durchfall oder Verstopfung	○	○	○
Schlafstörungen	○	○	○
Kopfschmerzen oder Gesichtsschmerzen	○	○	○
Unregelmässiger oder sehr schneller Herzschlag, starkes Herzklopfen	○	○	○
Schmerzen oder Druck im Brustbereich	○	○	○
Fieber	○	○	○
Schmerzen in den Schultern, im Nacken oder in den Armen	○	○	○

17 Haben Sie eine Krankheit oder Beschwerden schon seit 6 Monaten oder länger?

- ◯ Ja
- ◯ Nein
- ◯ Weiss nicht

18 Wenn Sie die letzte Frage mit „Ja“ beantwortet haben: Welche Krankheit oder Beschwerden sind das?

____

____

____

____

____

19 Wie ist Ihre Gesundheit allgemein?

- ◯ Sehr gut
- ◯ Gut
- ◯ Mittelmässig
- ◯ Schlecht
- ◯ Sehr schlecht
- ◯ Weiss nicht

20 Waren Sie in den letzten 6 Monaten durch Beschwerden eingeschränkt im Alltag?

- ◯ Ja, sehr
- ◯ Ja, etwas
- ◯ Nein
- ◯ Weiss nicht

21 Wie haben Sie sich in den letzten 4 Wochen gefühlt?

Kreuzen Sie bei allen Aussagen eine Antwort an.

	Immer	Meistens	Manchmal	Selten	Nie
Ich habe mich voller Leben gefühlt.	◯	◯	◯	◯	◯
Ich war sehr nervös.	◯	◯	◯	◯	◯
Ich war sehr niedergeschlagen.	◯	◯	◯	◯	◯
Ich war ruhig, ausgeglichen und gelassen.	◯	◯	◯	◯	◯
Ich war voller Energie.	◯	◯	◯	◯	◯
Ich war mutlos und traurig.	◯	◯	◯	◯	◯
Ich habe mich erschöpft gefühlt.	◯	◯	◯	◯	◯
Ich habe mich glücklich gefühlt.	◯	◯	◯	◯	◯
Ich war müde.	◯	◯	◯	◯	◯

22 **Wie oft hatten Sie diese Probleme in den letzten 2 Wochen?**
Kreuzen Sie bei allen Problemen eine Antwort an.

	Nie	Manchmal	Oft	Immer
Mir fehlte das Interesse und die Freude an allem.	○	○	○	○
Ich fühlte mich niedergeschlagen, traurig oder ohne Hoffnung.	○	○	○	○
Mein Schlaf war anders als sonst: Ich schlief schlechter oder mehr als sonst.	○	○	○	○
Ich war müde und schlapp.	○	○	○	○
Mein Appetit war anders als sonst: Ich hatte weniger oder viel mehr Appetit.	○	○	○	○
Ich fühlte mich wertlos, ich fühlte mich als Versager.	○	○	○	○
Ich konnte mich schlecht auf etwas konzentrieren, zum Beispiel beim Lesen.	○	○	○	○
Ich habe langsamer als sonst gesprochen und mich langsamer bewegt. Das ist auch anderen aufgefallen.	○	○	○	○
Ich war unruhig und wollte mich mehr bewegen als sonst.	○	○	○	○
Ich wollte am liebsten tot sein oder mir selber etwas antun.	○	○	○	○

23 Hat sich Ihr Gedächtnis in den letzten 12 Monaten verändert?

Erinnern Sie sich an Dinge besser oder weniger gut als vorher?

- ○ Ja
- ○ Nein
- ○ Weiss nicht

**24 Wurden Ihre kognitiven Fähigkeiten untersucht?
Zum Beispiel Ihre Fähigkeit, sich etwas zu merken? Oder etwas Neues zu lernen?**

- ○ Ja
- ○ Nein
- ○ Weiss nicht

25 Haben Sie in den letzten 7 Tagen ein Medikament eingenommen?

- ○ Ja
- ○ Nein

26 Wenn Sie die letzte Frage mit „Ja" beantwortet haben:

Welches Medikament haben Sie in den letzten 7 Tagen eingenommen?

Sie können mehrere Antworten ankreuzen.

- ◯ Medikament gegen Bluthochdruck
- ◯ Medikament gegen Herzbeschwerden
- ◯ Schlafmittel
- ◯ Schmerzmittel
- ◯ Beruhigungsmittel
- ◯ Medikament gegen Verstopfung
- ◯ Medikament gegen Asthma
- ◯ Medikament gegen zu hohes Cholesterin
- ◯ Medikament gegen Depression
- ◯ Medikament gegen Diabetes
- ◯ Medikament gegen Osteoporose
- ◯ Medikament zur Hormon-Behandlung
- ◯ Medikament gegen Epilepsie
- ◯ Andere Medikamente ______________________________
- ◯ Weiss nicht

27 Wie viele verschiedene Medikamente nehmen Sie täglich?

- ◯ 4 oder weniger
- ◯ 5 bis 10
- ◯ 11 oder mehr
- ◯ Weiss nicht

28 Wie oft trinken Sie alkoholische Getränke, zum Beispiel Bier, Wein oder Schnaps?

- ◯ 3 Mal pro Tag oder mehr
- ◯ 2 Mal pro Tag (zu Mahlzeiten)
- ◯ 1 Mal pro Tag
- ◯ Mehrere Male pro Woche
- ◯ 1 bis 2 Mal pro Woche
- ◯ 1 bis 3 Mal pro Monat
- ◯ Weniger als 1 Mal pro Monat
- ◯ Nie
- ◯ Weiss nicht

29 Rauchen Sie?

Wenn Sie nur selten rauchen, kreuzen Sie trotzdem „Ja" an.

- ◯ Ja
- ◯ Nein

30 Rauchen Sie täglich?

- ◯ Ja
- ◯ Nein

31 Wie gross sind Sie?

Grösse in cm: _______

- ◯ Weiss nicht

32 Wie schwer sind Sie?

Gewicht in kg: _______

- ◯ Weiss nicht

33 Wie oft treffen Sie sich mit Angehörigen bei Ihnen oder bei den anderen zu Hause?

- ◯ Täglich oder beinahe täglich
- ◯ Mindestens einmal pro Woche
- ◯ Mindestens einmal pro Monat
- ◯ Mindestens einmal pro Jahr
- ◯ Beinahe nie (weniger als einmal pro Jahr oder nie)
- ◯ Weiss nicht

34 Wie oft treffen Sie sich mit Freunden oder Bekannten bei Ihnen oder bei den anderen zu Hause?

- ◯ Täglich oder beinahe täglich
- ◯ Mindestens einmal pro Woche
- ◯ Mindestens einmal pro Monat
- ◯ Mindestens einmal pro Jahr
- ◯ Beinahe nie (weniger als einmal pro Jahr oder nie)
- ◯ Weiss nicht

Fragen zu Arztbesuchen und Spitalaufenthalten

35 Gehen Sie im Moment zum Arzt oder waren Sie innerhalb der letzten 12 Monate bei einem Arzt?

- ◯ Ja
- ◯ Nein
- ◯ Weiss nicht

36 Wenn Sie die vorhergehende Frage mit „Ja" beantwortet haben:

Warum sind oder waren Sie bei einem Arzt?

Sie können auch mehrere Antworten ankreuzen.

- ◯ Heuschnupfen oder andere Allergien
- ◯ Hoher Blutdruck
- ◯ Nierenkrankheit oder Nierensteine
- ◯ Chronische Bronchitis, andere Lungenprobleme
- ◯ Arthrose oder Rheuma
- ◯ Depression
- ◯ Migräne
- ◯ Osteoporose
- ◯ Anderes: ________________________________

37 Diese Frage ist nur für Frauen:

Wie oft waren Sie in den letzten 12 Monaten bei einem Frauenarzt oder einer Frauenärztin?

_____ Mal

- ◯ Weiss nicht

38 Waren Sie in den letzten 12 Monaten in Behandlung wegen einem psychischen Problem?

- ◯ Nein
- ◯ Ja, ____ Mal.
- ◯ Weiss nicht

39 Waren Sie in den letzten 12 Monaten geplant in einem Spital oder einer speziellen Klinik, ohne dass Sie übernachten mussten? Kuraufenthalte zählen nicht.

- ◯ Nein
- ◯ Ja, ____ Tage.
- ◯ Weiss nicht

40 Waren Sie in den letzten 12 Monaten geplant in einem Spital oder einer Spezialklinik und mussten dort übernachten?

- ◯ Nein
- ◯ Ja, ____ Übernachtungen
- ◯ Weiss nicht

41 Waren Sie in den letzten 12 Monaten als Notfall in einem Spital?

- ◯ Nein
- ◯ Ja, ____ Tage.
- ◯ Weiss nicht

42 Wenn Sie die letzte Frage mit „Ja“ beantwortet haben:

Was war der Grund für den Notfall?

Sie können auch mehrere Antworten ankreuzen.

- ◯ Unfall oder Verletzung
- ◯ Eine neue Erkrankung, neue Symptome
- ◯ Eine Verschlechterung von bekannten Erkrankungen und Symptomen
- ◯ Anderes ______________________________
- ◯ Weiss nicht

43 Wenn Sie bei einem Arzt oder einer Ärztin sind, können Sie sich gut verständigen? Versteht der Arzt oder die Ärztin Ihr Anliegen?

- ◯ Immer
- ◯ Meistens
- ◯ Manchmal
- ◯ Selten
- ◯ Nie
- ◯ Weiss nicht

44 Wie oft hat im Spital eine Person zwischen Ihnen und dem Arzt oder der Ärztin übersetzt, damit Sie sich besser verständigen konnten?

- ◯ Immer
- ◯ Meistens
- ◯ Manchmal
- ◯ Selten
- ◯ Nie
- ◯ Weiss nicht

45 Wer hat Sie unterstützt, wenn Sie nicht verstanden wurden?

Sie können auch mehrere Antworten ankreuzen.

- ◯ Ein ausgebildeter Dolmetscher oder eine Dolmetscherin
- ◯ Eine Betreuungsperson oder eine andere Fachperson aus der Heilpädagogik oder Sozialpädagogik
- ◯ Eine Fachperson vom Spital
- ◯ Zufällig anwesende Personen
- ◯ Verwandte, Freunde oder Bekannte
- ◯ Niemand
- ◯ Weiss nicht

46 Welche dieser Untersuchungen haben Sie schon einmal gemacht?

- ◯ Bei Männern: Prostata-Untersuchung
- ◯ Bei Frauen: Mammographie, also Röntgen der Brust
- ◯ Untersuchung des Stuhls
- ◯ Darmspiegelung, also Untersuchung des Darms mit einer Kamera
- ◯ Weiss nicht

Haben Sie diesen Fragebogen allein ausgefüllt oder hat Ihnen jemand geholfen?

- ◯ Ich habe den Fragebogen alleine ausgefüllt.
- ◯ Jemand hat mir geholfen.

Wir möchten diese Umfrage in einem Jahr noch einmal machen.

Dürfen wir Ihnen diesen Fragebogen wieder schicken?

- ◯ Ja
- ◯ Nein

Vielen Dank, dass Sie diesen Fragebogen ausgefüllt haben!

Bitte senden Sie den Fragebogen in den nächsten zwei Wochen an uns zurück. Verwenden Sie den beiliegenden Umschlag.

Bei Fragen können Sie sich jederzeit an Monika Wicki wenden:

Monika T. Wicki
Interkantonale Hochschule für Heilpädagogik (HfH) Zürich
Schaffhauserstrasse 239
Postfach 5850
Telefon: 044 317 12 34
E-Mail: monika.wicki@hfh.ch
