# Supplementary material for: Physical and mental health of older people with disabilities in residential homes in Switzerland
Source: SAGE Open Med. 2021 Mar 10;9:20503121211000530. doi: 10.1177/20503121211000530 (PMC7958166; doi:10.1177/20503121211000530)
Supplement: sj-pdf-2-smo-10.1177_20503121211000530 – Supplemental material for Physical and mental health of older people with disabilities in residential homes in Switzerland [file sj-pdf-2-smo-10.1177_20503121211000530.pdf]

Interkantonale Hochschule für Heilpädagogik

SAMW ASSM
Schweizerische Akademie der Medizinischen Wissenschaften
Académie Suisse des Sciences Médicales
Accademia Svizzera delle Scienze Mediche
Swiss Academy of Medical Sciences

STANLEY THOMAS JOHNSON STIFTUNG

# Health questionnaire


Dr. Monika T. Wicki
Dozentin
Interkantonale Hochschule für Heilpädagogik Zürich
Schaffhauserstrasse 239
Postfach 5850
CH-8050 Zürich

T +41 (0)44 317 12 34
F +41 (0)44 317 11 10
monika.wicki@hfh.ch
www.hfh.ch

## Socio-demographic information

**1 Date of birth**

Day:____ Month:__________ Year:____

**2 Sex**

◯ man

◯ woman

**3 Where have you been born?**

◯ In Switzerland

◯ Not in Switzerland

◯ Don’t know

**4 Which language is your first language?**

◯ German

◯ French

◯ Italian

◯ Rhaeto-Romanic

◯ Other language which:___________

◯ Don’t know

Schaffhauserstrasse 239
Postfach 5850
CH-8050 Zürich

T +41 44 317 12 34
F +41 44 317 11 10

wicki.monika@hfh.ch
www.hfh.ch

**5 What is the highest education you have completed?**

◯

◯

◯

◯ ◯ High school diploma

- ◯ Compulsory schooling has not been completed
- ◯ Compulsory schooling completed
- ◯ EF Swiss Federal Certificate of Competence EFZ (or equivalent)
- ◯ Federal professional certificate EBA (or equivalent)
- ◯ Training financed by the disability insurance (e.g. practical training according to Insos)
- ◯ High school diploma
- ◯ technical middle school (or equivalent)
- ◯ Federal diploma or specialist certificate (or equivalent)
- ◯ Higher technical school (or equivalent)
- ◯ University degree
- ◯ Don't know

**Information on the living and support situation**

**6 Where do you live?**

◯ In my own apartment

◯ With relatives

◯ In a residential facility for people with disabilities

◯ In a nursing home

◯ Other place where: _______________

**7 Do you have a steady partner?**

◯ Yes

◯ No

## Information on the disability pension and the need for support

**12** **You get this questionnaire because you have an IV pension. What is the reason for the pension? (You can also tick several answers here)**

- ◯ physical disability
- ◯ psychological disability
- ◯ intellectual disability
- ◯ other ___________________________________
- ◯ Don’t know

**13** **How long have you had the disability pension?**

Number of years _____ (Year of the first pension ________)

**14** **Please tick each of the following activities which applies to you. You can:**

| | Yes, without difficulties | Yes, with minor difficulties | yes, with severe difficulty | I can't achieve it by myself |
|---|---|---|---|---|
| Feeding yourself | ◯ | ◯ | ◯ | ◯ |
| Getting in and out of a bed or chair | ◯ | ◯ | ◯ | ◯ |
| Dressing and undressing | ◯ | ◯ | ◯ | ◯ |
| Using toilets | ◯ | ◯ | ◯ | ◯ |
| Bathing or showering | ◯ | ◯ | ◯ | ◯ |

**15 Please tick each of the following activities which applies to you. You can:**

| | Yes, without difficulties | Yes, with minor difficulties | yes, with severe difficulty | I can't achieve it by myself |
|---|---|---|---|---|
| Preparing meals | ◯ | ◯ | ◯ | ◯ |
| Using the telephone | ◯ | ◯ | ◯ | ◯ |
| Shopping | ◯ | ◯ | ◯ | ◯ |
| Wash laundry | ◯ | ◯ | ◯ | ◯ |
| Light housework | ◯ | ◯ | ◯ | ◯ |
| Occasional heavy housework | ◯ | ◯ | ◯ | ◯ |
| Taking care of finances | ◯ | ◯ | ◯ | ◯ |
| Use public transport | ◯ | ◯ | ◯ | ◯ |

Schaffhauserstrasse 239
Postfach 5850
CH-8050 Zürich

T +41 44 317 12 34
F +41 44 317 11 10

wicki.monika@hfh.ch
www.hfh.ch

## Angaben zum Gesundheitszustand

**16 Have you had any or all of the following symptoms in the past four weeks? Check what applies to you:**

| | Not at all | Some | Strong |
|---|---|---|---|
| Back or lower back pain | ◯ | ◯ | ◯ |
| General weakness, tiredness, lack of energy | ◯ | ◯ | ◯ |
| Pain or feeling of pressure in the abdomen | ◯ | ◯ | ◯ |
| Diarrhea, constipation or both | ◯ | ◯ | ◯ |
| Difficulty falling asleep or staying asleep | ◯ | ◯ | ◯ |
| Headache, pressure in the head or facial pain | ◯ | ◯ | ◯ |
| Palpitations, rapid heartbeat or stumbling | ◯ | ◯ | ◯ |
| Pain or pressure in the chest area | ◯ | ◯ | ◯ |
| Fever | ◯ | ◯ | ◯ |
| Pain in the shoulders, neck and / or arms | ◯ | ◯ | ◯ |

**17 Do you have any longstanding illness or [longstanding]health problem? [By longstanding I mean illnesses or health problems which have lasted, or are expected to last, for 6 months or more].**

◯ Yes

◯ No

◯ Don't know

**19 How is your health in general? Is it...**

- ◯ very good
- ◯ good
- ◯ fair
- ◯ bad
- ◯ very bad
- ◯ don’t know

**20 For at least the past 6 months, to what extent have you been limited because of a health problem in activities people usually do? Would you say you have been**

- ◯ severely limited
- ◯ limited but not severely
- ◯ not limited at all
- ◯ don’t know

**21 How much of the time, during the past weeks...**

| | All of the time | Most of the time | Some of the time | A little of the time | None of the time |
|---|---|---|---|---|---|
| Did you feel full of life? | ◯ | ◯ | ◯ | ◯ | ◯ |
| Have you been very nervous? | ◯ | ◯ | ◯ | ◯ | ◯ |
| Have you felt so down in the dumps that nothing could cheer you up? | ◯ | ◯ | ◯ | ◯ | ◯ |
| Have you felt calm and peaceful? | ◯ | ◯ | ◯ | ◯ | ◯ |
| Did you have a lot of energy? | ◯ | ◯ | ◯ | ◯ | ◯ |
| Have you felt down-hearted and depressed? | ◯ | ◯ | ◯ | ◯ | ◯ |
| Did you feel worn out? | ◯ | ◯ | ◯ | ◯ | ◯ |
| Have you been happy? | ◯ | ◯ | ◯ | ◯ | ◯ |
| Did you feel tired? | ◯ | ◯ | ◯ | ◯ | ◯ |

**22** **Over the last 2 weeks, how often have you been bothered by any of the following problems?**

| | Not at all | Several days | More than half the days | Nearly every day |
|---|---|---|---|---|
| Little interest or pleasure in doing things | ◯ | ◯ | ◯ | ◯ |
| Feeling down, depressed, or hopeless | ◯ | ◯ | ◯ | ◯ |
| Trouble falling or staying asleep, or sleeping too much | ◯ | ◯ | ◯ | ◯ |
| Feeling tired or having little energy | ◯ | ◯ | ◯ | ◯ |
| Poor appetite or overeating | ◯ | ◯ | ◯ | ◯ |
| Feeling bad about yourself — or that you are a failure or have let yourself or your family down | ◯ | ◯ | ◯ | ◯ |
| Trouble concentrating on things, such as reading the newspaper or watching television | ◯ | ◯ | ◯ | ◯ |
| Moving or speaking so slowly that other people could have noticed? Or the opposite — being so fidgety or restless that you have been moving around a lot more than usual | ◯ | ◯ | ◯ | ◯ |
| Thoughts that you would be better off dead or of hurting yourself in some way | ◯ | ◯ | ◯ | ◯ |

**23** **Have you noticed changes in your memory in the past 12 months?**

◯ Yes

◯ No

◯ Don’t know

**24 Have you ever had an exam to assess your cognitive status?**

- ◯ Yes
- ◯ No
- ◯ Don’t know

**25 Haben Sie in den letzten 7 Tagen irgendein Medikament genommen?**

- ◯ Ja
- ◯ Nein

**26**

**Please check:**

- ◯ have the questionnaire filled out in whole or in part with the help of another person
- ◯ I completed the questionnaire alone
